# Protection against lethal sepsis following immunization with *Candida* species varies by isolate and inversely correlates with bone marrow tissue damage

**DOI:** 10.1128/iai.00252-23

**Published:** 2023-09-13

**Authors:** Elizabeth A. Lilly, Breah E. Bender, Mairi C. Noverr, Paul L. Fidel

**Affiliations:** 1 Department of Microbiology and Immunology, School of Medicine, Tulane University, New Orleans, Louisiana, USA; 2 Center of Excellence in Oral and Craniofacial Biology, Louisiana State University Health Sciences Center School of Dentistry, New Orleans, Louisiana, USA; University of California, Davis, Davis, California, USA

**Keywords:** intra-abdominal infection, polymicrobial infection, *Candida*, sepsis, bone marrow

## Abstract

Protection against lethal *Candida albicans* (*Ca*)/*Staphylococcus aureus* (*Sa*) intra-abdominal infection (IAI)-mediated sepsis can be achieved by a novel form of trained innate immunity (TII) involving Gr-1+ myeloid-derived suppressor cells (MDSCs) that are induced by inoculation (immunization) with low virulence *Candida* species [i.e., *Candida dubliniensis* (*Cd*)] that infiltrate the bone marrow (BM). In contrast, more virulent *Candida* species (i.e., *C. albicans*), even at sub-lethal inocula, fail to induce similar levels of protection. The purpose of the present study was to test the hypothesis that the level of TII-mediated protection induced by *Ca* strains inversely correlates with damage in the BM as a reflection of virulence. Mice were immunized by intraperitoneal inoculation with several parental and mutant strains of *C. albicans* deficient in virulence factors (hyphal formation and candidalysin production), followed by an intraperitoneal *Ca/Sa* challenge 14 d later and monitored for sepsis and mortality. Whole femur bones were collected 24 h and 13 d after immunization and assessed for BM tissue/cellular damage via ferroptosis and histology. While immunization with standard but not sub-lethal inocula of most wild-type *C. albicans* strains resulted in considerable mortality, protection against lethal *Ca*/*Sa* IAI challenge varied by strain was usually less than that for *C. dubliniensis*, with no differences observed between parental and corresponding mutants. Finally, levels of protection afforded by the *Ca* strains were inversely correlated with BM tissue damage (*R* ^2^ = −0.773). TII-mediated protection against lethal *Ca/Sa* sepsis induced by *Candida* strain immunization inversely correlates with BM tissue/cellular damage as a reflection of localized virulence.

## INTRODUCTION

Intra-abdominal infections (IAI) can occur as a result of bowel perforations, laparotomy surgery, intestinal hernias, and insertion of medical devices, such as peritoneal catheters (1, 2). If these infections are left untreated or misdiagnosed, microorganisms can migrate into the bloodstream, causing sepsis and leading to significant morbidity and mortality (3 – 5). IAIs are often polymicrobial and infections involving both fungal and bacterial pathogens result in significantly higher mortality rates compared with infections involving bacterial species alone (6 – 12). Along with gram-negative enteric bacteria, gram-positive species including *Staphylococcus aureus* (*Sa*) are also co-isolated pathogens, particularly with nosocomial infections (13 – 18). This polymicrobial pairing can be enhanced based on a predilection of *Sa* for *Candida albicans* (*Ca*) hyphae, which are observed co-associated within peritoneal tissue lesions (19). The pathogenesis of this lethal polymicrobial IAI is becoming better understood, with inflammatory responses leading to sepsis considered a key hallmark factor in mortality (20, 21).

Our laboratory has been studying *Ca/Sa* polymicrobial IAI using an experimental mouse model that results in 80–90% mortality by 48 to 72 h post-inoculation (22 – 24). Characterization of host responses during *Ca/Sa* polymicrobial IAI revealed that mortality is associated with robust inflammation, with elevated levels of hallmark sepsis proinflammatory cytokines (IL-6, TNF-α, and IL-1β), both locally and systemically, as early as 4 h and continuing through 24 to 48 h post-inoculation. On the other hand, at similar time points there were equivalent microbial burdens in non-lethal monomicrobial and lethal polymicrobial infections, both locally in the peritoneal cavity, as well as in adjacent (spleen and kidney) and non-adjacent organs (brain), indicating that robust inflammation (sepsis) is the key factor in the lethal outcome (24).

Subsequent studies investigated whether all *Candida* species that cause human disease also lead to synergistic mortality during polymicrobial infection. IAI inoculation with *Sa* and a variety of non-*albicans Candida* (NAC) species resulted in varying levels of mortality. Co-infections with *Candida glabrata* (*Cg*) or *Candida dubliniensis* (*Cd*) and *Sa* result in no mortality, whereas co-infections with *Candida krusei* (*Ck*) or *Candida tropicalis* (*Ct*) and *Sa* lead to 80–90% mortality (25). As with *Ca*, monomicrobial infections with the majority of these *Candida* species or *Sa* alone were not lethal out to 5–10 d post-inoculation (23).

We have also shown that protection against *Ca/Sa* lethal sepsis can be achieved by prior immunization [intraperitoneal (i.p.) or i.v.] with low virulence *Candida* species, including *Cd, Cg, Candida auris*, and *Ca* efg1Δ/Δ cph1Δ/Δ mutant (26). In each case, immunization followed by a lethal *Ca/Sa* challenge 14 d later resulted in >80% survival. Protection was long-lived (up to 60 d post-challenge) and mediated primarily by Gr-1+ polymorphonuclear leukocytes of the innate response, rather than adaptive immunity (25, 26). This innate-mediated protection was suggestive of a form of trained innate immunity (TII). TII was first described in macrophages “trained” by epigenetic reprogramming leading to enhanced responsiveness to secondary infection (27). Subsequent studies in our laboratory showed that the Gr-1+ cells mediating protection were long-lived myeloid-derived suppressor cells (MDSCs) (28) which have been reported in other models of sepsis and in patients with candidiasis (29, 30). MDSCs are induced and expanded in the bone marrow (BM) (31 – 33). We have further shown that the *Candida* species used for the primary immunization access the BM within 24 h consistent as a requirement for MDSC development (26).

Interestingly, immunization with more virulent *Candida* species (*Ck, Ct,* and *Ca*), even at lower inocula that spared any initial mortality over a 14-d period, resulted in much lower protection against lethal sepsis compared to the low virulent species (40–50% vs 80–100%, respectively), despite similar infiltration into the BM (25, 26). We hypothesize that the induction of the protective MDSC response is inversely proportional to the level of tissue damage caused by the *Candida* species in the BM. The purpose of the present study was to test this hypothesis by comparing protection induced by a variety of *Ca* isolates with varying levels of reported virulence in other models of infection (24, 34 – 39). Accordingly, we employed several *Ca* strains that were clinical isolates (529L) or mutant strains deficient in one or more virulence factors (TNRG1, efg1Δ/Δ cph1Δ/Δ, and ece1Δ/Δ) together with their isogenic parent strains (TT21, DAY185, and BWPI7 + CIp30), to correlate their ability to protect against lethal sepsis, with immunization-associated tissue damage in the BM.

## MATERIALS AND METHODS

### Mice

Female Swiss Webster mice, 5 to 7 weeks of age, were purchased from Charles River Laboratories. Animals were housed and handled according to institutionally recommended guidelines. All experiments involving animals were approved by the Tulane University School of Medicine Institutional Animal Care and Use Committee (IACUC).

### Strains and growth conditions

*C. albicans* strain DAY185, a complemented prototroph derived from a triple auxotrophic strain (BWP17; parent, SC5314) was a gift from Dr. Aaron Mitchell (Carnegie Melon University, Pittsburgh, PA). The *C. dubliniensis* wild-type strain (Wü284) was kindly provided by Dr. Gary Moran (Trinity College, Dublin, Ireland). Yeast-locked strain (TNRG1), the corresponding wild-type strain (TT21), as well as *C. albicans* mutant efg1Δ/Δ cph1Δ/Δ were provided by Glen Palmer (University of Tennessee Health Science Center, Memphis, TN). The clinical oral isolate, 529L, as well as the ece1Δ/Δ mutant and corresponding wild-type strain (BWPI7 + CIp30) were generously donated by Julian Naglik (King’s College London, UK). All frozen stocks were maintained at −80°C and streaked onto yeast peptone dextrose (YPD) agar prior to use. A single colony was transferred to 10 mL of YPD broth and shaken at 30°C for 12–18 h. The methicillin-resistant *Sa* strain NRS383 used was obtained from the Network on Antimicrobial Resistance in *Sa* (NARSA) data bank. Frozen stocks were maintained at −80°C and streaked onto Trypticase soy agar (TSA) prior to use. A single colony was transferred to 10 mL of Trypticase soy broth (TSB) and shaken at 37°C overnight. On the following day, the overnight culture was diluted 1:100 in fresh TSB and shaken at 37°C for 3 h until the culture reached the log phase of growth. Prior to inoculation, organisms were washed three times by centrifugation in sterile phosphate-buffered saline (PBS; pH 7.4), counted on a hemocytometer, and diluted in sterile PBS to prepare standardized inocula. To visualize cells, *Sa* was stained with LIVE/DEAD BacLight Bacterial Viability Kit (Invitrogen, Waltham, MA) 5 min prior to counting.

### Murine model of fungal-bacterialintra-abdominal infection

(*i*) *Immunization*. Groups (*n* = 10) of 6-week-old female mice were injected with the various *Candida* strains intraperitoneally (i.p.) at the standard (1.75 × 10^7^) or sub-lethal (1 × 10^6^) inocula in a volume of 200 µL of sterile PBS 14 d prior to lethal challenge.

(*ii*) *Lethal Challenge*. Mice were injected i.p. with a lethal challenge of *Ca* (1.75 × 10^7^/mouse) and *Sa* (8 × 10^7^/mouse) in a volume of 200 µL of sterile PBS and observed for morbidity (hunched posture, inactivity, and ruffled fur) and mortality up to 10 d after re-challenge. Mice who reached clinical endpoints prior to the study endpoint were humanely euthanized following IACUC-approved euthanasia procedures. Of note, previous studies evaluating sex as a biological variable, using *C. dubliniensis* as the immunizing strain together with Ca/Sa lethal challenge, showed similar levels of protection against lethal challenge and median day of death in lethal challenge controls in males and females (Noverr, unpublished data). Hence, female mice were used exclusively in the present study.

### Histological analysis of whole femur for evidence of tissue damage

(*i*) *Bone architecture by Periodic Acid-Schiff* (*PAS*). One or 13 d after immunization, mice (*n* = 5/group) were euthanized and whole femurs were excised (40). Bones were cleaned of all soft tissue, placed in tissue cassettes, and immersed in 10% neutral buffered formalin. After 7 d, selected bones (24 h post-immunization) were sent for histological preparation (paraffin-embedding, sectioning, deparaffinization, and rehydration) and stained using the standard Periodic Acid-Schiff staining technique (GNO Histology Consultants, LLC, Marrero, LA).

(*ii*) *Ferroptosis by 4-HNE staining*. Unstained 24 h or 13 d post-immunization bone sections underwent histological preparation as described above. 4-HNE tissue staining was performed as described previously (41). Briefly, antigen retrieval was immediately performed using a citrate-based Antigen Unmasking Solution (Vector Laboratories Inc., Burlingame, CA) heated to 95°C. Slides were placed in the pre-heated solution for 2 min and then rinsed with distilled water followed by PBS. Sections were blocked using 1% BSA in PBST (PBS + 0.1% Tween-20) for 30 min at room temperature. Excess liquid was drained, and sections were incubated with goat anti-mouse 4-HNE antibody (ab46545; Abcam, Boston, MA) overnight at 4°C in a humidified chamber. Slides were washed three times in PBS (5 min/wash) followed by incubation with conjugated donkey anti-goat IgG secondary antibody (Alexa Fluor 488; Abcam) for 1 h at room temperature in the dark. Slides were washed three times in PBS (5 min/wash) followed by mounting/counterstaining with DAPI-Aqueous Mounting Media with Fluoroshield (Abcam). All tissue images were captured with the Lionheart FX Automated Microscope (BioTek Instruments, Winooski, VT) using the same image acquisition settings for each slide. Mean fluorescence intensity of each image was calculated using Gen5 microscopy and imaging software (BioTek).

### Statistics

All statistical analyses were performed using Prism software (GraphPad, San Diego, CA). Survival curves were compared using the log-rank (Mantel-Cox) test. Mean fluorescence intensities of 4-HNE were analyzed by Student’s *t* test. The Pearson product-moment correlation coefficient (*R* ^2^, *P* value) was calculated to determine correlations. Significance was defined as *P* < 0.05.

## RESULTS

### Immunization inocula results in varying levels of mortality among *C. albicans* strains

**Fig 1:**
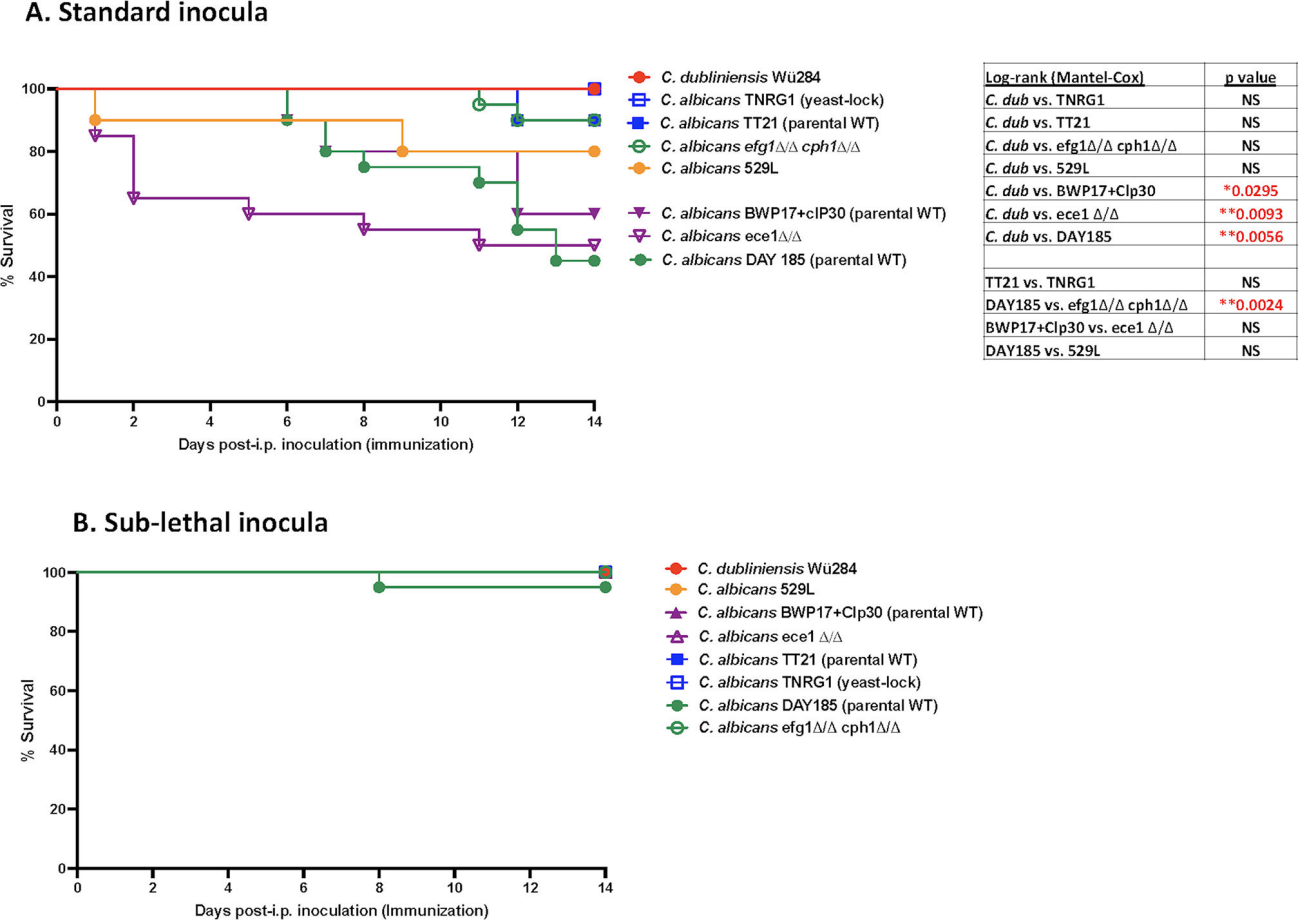
Immunization inocula results in varying levels of mortality among *C. albicans* strains. Mice (*n* = 10/group) were given inocula defined as standard (1.75 × 10^7^) or sub-lethal (1 × 10^6^) as an intraperitoneal inoculation (immunization) of *C. dubliniensis* wild-type strain (Wü284), *C. albicans* wild-type strains (DAY185, BWP17 + Clp30, TT21, and 529L), or corresponding mutant strains (efg1Δ/Δ cph1Δ/Δ, ece1Δ/Δ, and TNRG1), and monitored for survival over 14 d (standard period of time prior to lethal challenge). (**A**) Percentage survival of mice given the standard inocula. (**B**) Percentage survival of mice given the sub-lethal inocula. Results shown are cumulative of four independent experiments. Data were analyzed using the log-rank (Mantel-Cox) test. ****P < 0.0001; ***P < 0.001; **P < 0.01; and *P < 0.05 (significance as compared to *C. dubliniensis* protection control between mutant and corresponding wild-type). Actual *P* values are listed in tables. WT, wild-type.

Building on the observation that i.p. immunization with several virulent *Candida* species resulted in varying levels of mortality and/or low-level protection against lethal *Ca/Sa* challenge, we sought to determine the mortality following inoculation with *Ca* species of varying levels of reported virulence (in other models of infection) (36 – 39, 42 – 52). The first series of studies employed the standard inocula (1.75 × 10^7^) used for *Cd* immunization in previous studies (25, 26, 53) for a number of *Ca* wild-type and isogenic mutant strains. Survival results are shown in Fig. 1A. Along with control strains exhibiting little to no mortality (*C. dubliniensis* Wü284 and *C. albicans* efg1Δ/Δ cph1Δ/Δ mutant), inoculation with *C. albicans* yeast-lock mutant (TNRG1) and corresponding parental strain (TT21) similarly resulted in little to no mortality (100% and 90% survival, respectively). Inoculation with the clinical oral wild-type isolate, 529L, resulted in 20% mortality (~80% survival). However, inoculation with *C. albicans* strain DAY185 (parental strain for *C. albicans* efg1Δ/Δ cph1Δ/Δ mutant), parental strain BWP17 + clP30, and isogenic ece1Δ/Δ mutant, which is less virulent in mucosal and systemic infection models (39, 42 – 44), all resulted in intermediate levels of mortality (40–60% survival). Therefore, we tested “sub-lethal” (1 × 10^6^) inocula previously established for other virulent NAC species (22, 24). Results in Fig. 1B show little to no mortality following inoculation of all *C. albicans* strains tested.

### Protection against lethal challenge (*Ca*/*Sa*) by *C. albicans* immunization varies by isolate

**Fig 2:**
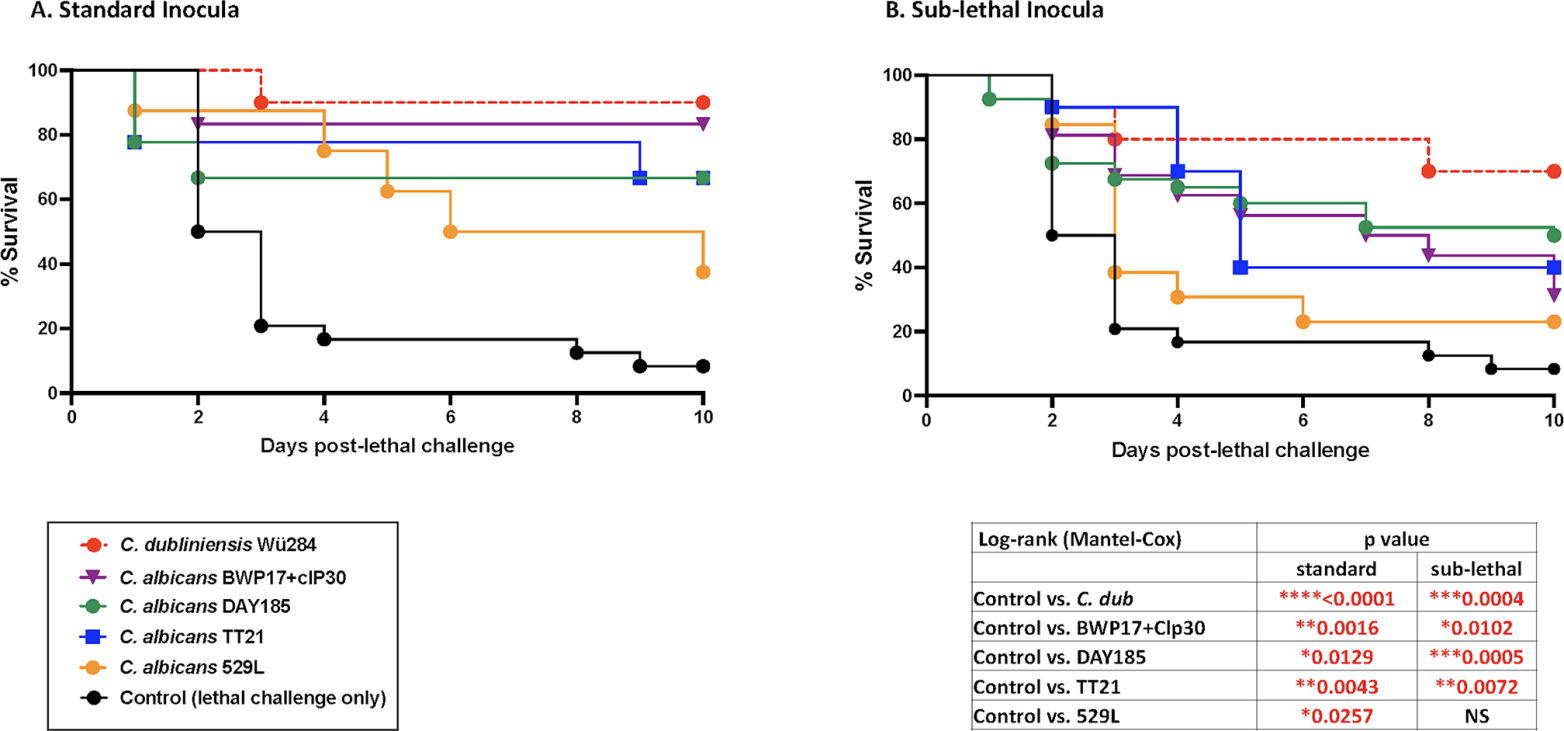
Protection against lethal challenge (*Ca*/*Sa*) following immunization with wild-type *C. albicans* varies by immunization strain. Mice (*n* = 10/group) were given a standard (1.75 × 10^7^) or sub-lethal (1 × 10^6^) intraperitoneal inoculation (immunization) of *C. albicans* wild-type strains (DAY185, BWP17 + clP30, TT21, and 529L) followed 14 d later by lethal challenge (1.75 × 10^7^ *Ca* + 8 × 10^7^ *Sa*). Mice were monitored for sepsis and mortality for 10 d post-lethal challenge. (**A**) Percentage survival of mice given the standard inocula immunization (**B**) Percentage survival of mice given the sub-lethal inocula immunization. *C. dubliniensis* wild-type strain (Wü284) at the standard/sub-lethal inocula was included as a positive control for protection. Animals receiving no immunization served as the negative (lethal) control. Results shown are cumulative of four independent experiments. Data were analyzed using the log-rank (Mantel-Cox) test. ****P < 0.0001; ***P < 0.001; **P < 0.01; and *P < 0.05 (significance as compared to lethal challenge control). Actual *P* values are listed in tables. WT, wild-type

We next tested the protective potential of the various wild-type *C. albicans* isolates against lethal IAI challenge. Following the 14-day immunization period with either standard or sub-lethal inocula, surviving mice were injected i.p. with a lethal challenge of *Ca* (1.75 × 10^7^/mouse) and *Sa* (8 × 10^7^/mouse) and observed for morbidity (hunched posture, inactivity, and ruffled fur) and mortality over a period of 10 d. Immunization with *Cd* was used as a positive control for strong protection. Compared to protection elicited by *Cd* immunization (standard or sub-lethal inocula), immunization with all wild-type *Ca* isolates resulted in lower levels of protection, with sub-lethal inocula generally resulting in lower levels of protection than that observed in surviving mice given the standard inocula (20–60% vs 40–80%; Fig. 2A and B). Despite these differences, all strains induced significant protection compared to lethal challenge control, with the exception of *C. albicans* 529L at the sub-lethal inocula immunization. Interestingly, *C. albicans* 529L exhibited the lowest level of protection at both the standard and sub-lethal inocula immunizations (40% and 20%, respectively). No significant differences in levels of protection were detected between the various isolates, both at the standard or sub-lethal inocula.

### *C. albicans* mutant strain defects have little effect on protection against lethal challenge (*Ca*/*Sa*)

**Fig 3:**
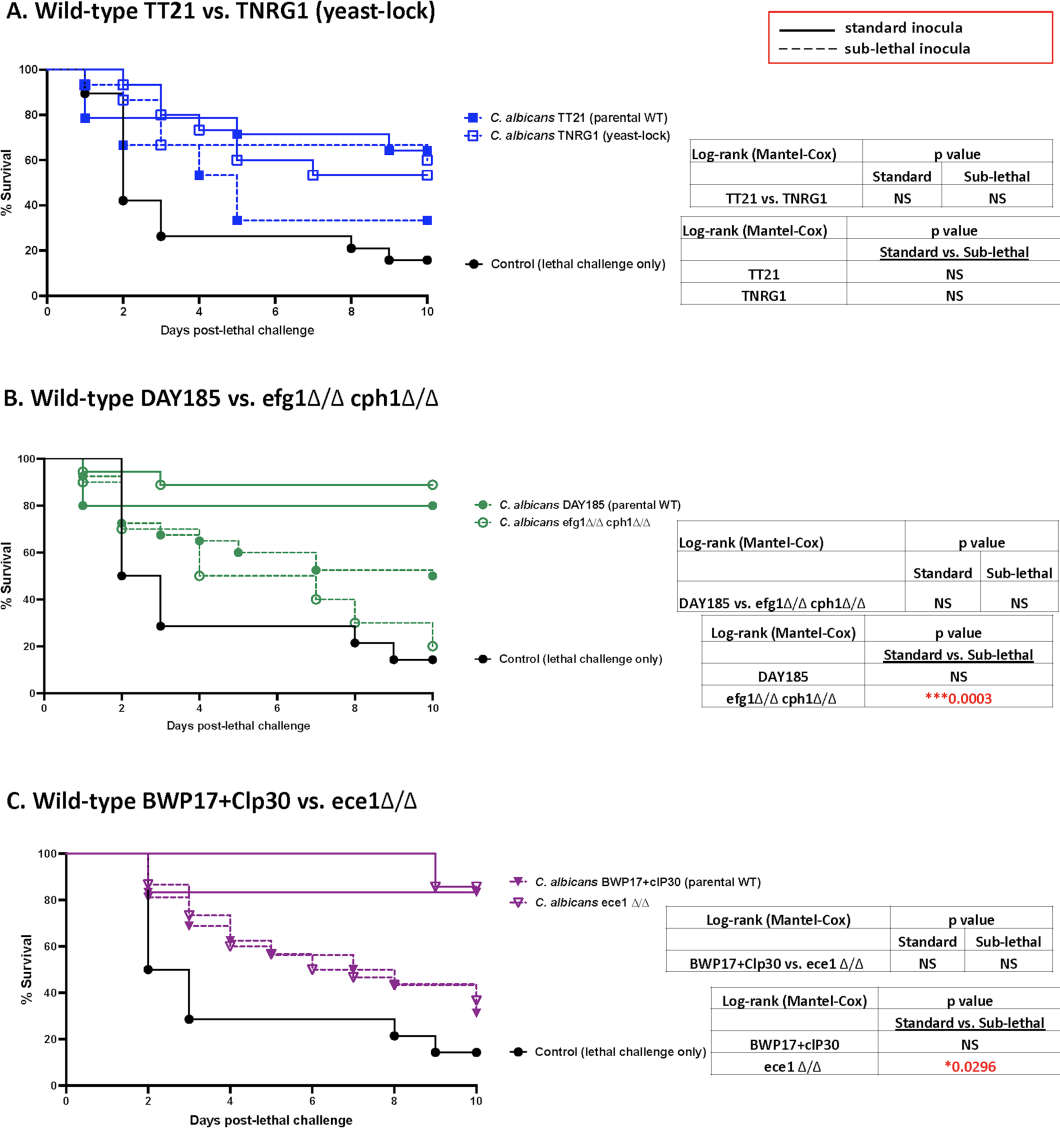
Immunization with *C. albicans* wild-type and mutant strain pairs result in similar levels of protection against lethal challenge (*Ca*/*Sa*). Mice (*n* = 10/group) were given a standard (1.75 × 10^7^) or sub-lethal (1 × 10^6^) intraperitoneal inoculation (immunization) of *C. albicans* wild-type strains (DAY185, BWP17 + clP30, and TT21) or corresponding mutant strains (efg1Δ/Δ cph1Δ/Δ, ece1Δ/Δ, and TNRG1) followed 14 d later by lethal challenge (1.75 × 10^7^ *Ca* + 8 × 10^7^ *Sa*). Mice were monitored for sepsis and mortality for 10 d post-lethal challenge. (**A**) Parental wild-type *C. albicans* TT21 vs TNRG1 (yeast-lock) (**B**) Parental wild-type *C. albicans* DAY185 vs efg1Δ/Δ cph1Δ/Δ (**C**) Parental wild-type *C. albicans* BWP17 + Clp30 vs ece1Δ/Δ. Animals receiving no immunization served as the negative (lethal) control. Results shown are cumulative of six independent experiments. Data were analyzed using the log-rank (Mantel-Cox) test. ****P < 0.0001; ***P < 0.001; **P < 0.01; and *P < 0.05 (significance as compared between strains and between inocula). Actual *P* values are listed in tables. WT, wild-type

We next compared the relative levels of protection between each wild-type and isogenic mutant strain at both standard and sub-lethal immunizing inocula. For immunization with wild-type *Ca* TT21 and yeast-lock mutant TNRG1, a range of partial protection (30–70%) was observed at either inocula (Fig. 3A) with no significant differences observed between wild-type and mutant or between standard or sub-lethal inocula of each isolate. In the case of immunization with wild-type parental *Ca* DAY185 and corresponding efg1Δ/Δ cph1Δ/Δ mutant, the standard inocula conferred 80% and 90% protection, respectively, while immunization with the sub-lethal inocula resulted in lower levels of protection (50% and 20%, respectively) but no significant differences between wild-type and mutant or between the wild-type at standard or sub-lethal inocula. A significantly lower level of protection was observed for the mutant strain at the sub-lethal compared to the standard inocula (*P* = 0.0003) (Fig. 3B). Similarly, for immunization with either *Ca* wild-type strain BWP17 + Clp30 or the corresponding mutant ece1Δ/Δ, standard inocula immunization with either isolate resulted in 80% protection, while sub-lethal inocula resulted in lower levels of protection (40% and 30%, respectively) but with no significant differences between wild-type and mutant or between the wild-type at standard or sub-lethal inocula. A significantly lower level of protection was observed for the mutant strain at the sub-lethal compared to the standard inocula (*P* = 0.030) (Fig. 3C). Included in these experiments were an additional set of mice (*n* = 4/group) given standard or sub-lethal inoculations of wild-type/parental strains (*C. albicans* DAY185; BWP17 + Clp30), including the isogenic mutant strains (*C. albicans* efg1Δ/Δ cph1Δ/Δ; ece1Δ/Δ), to confirm bone marrow infiltration at 24 h post-immunization. While there were consistently lower levels of infiltration with sub-lethal inocula, there were no statistically significant differences between standard and sub-lethal inocula for each isolate or between isolates, with the exception of standard vs sub-lethal inocula for *C. albicans* DAY185 (*P* = 0.015) (Fig. S1).

### Ferroptosis in bone marrow post-immunization with *Candida* strains as a measure of tissue damage

**Fig 4:**
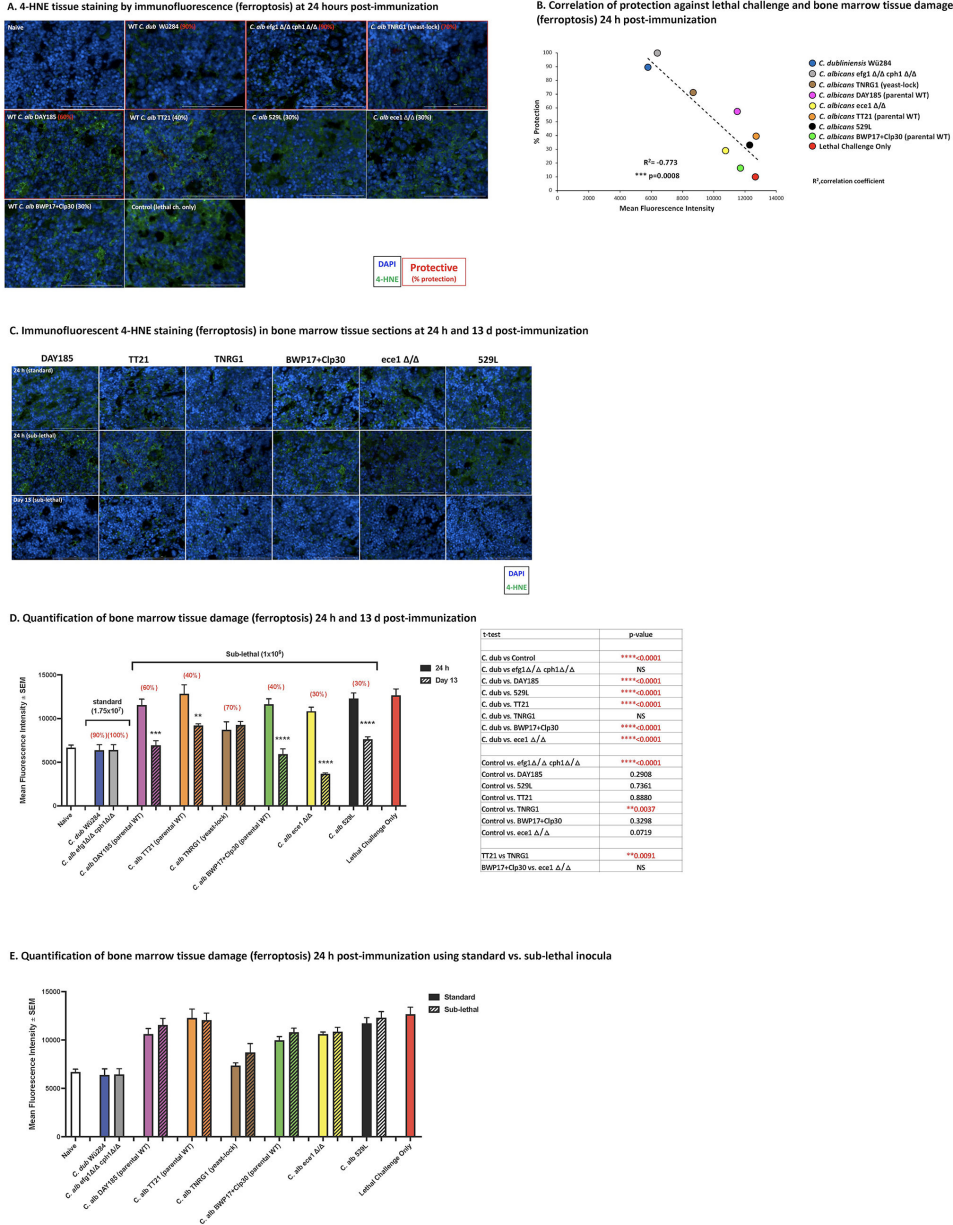
Ferroptosis in bone marrow 24 h post-immunization with *Candida* strains as a measure of tissue damage. Mice (*n* = 5) were injected i.p. with the standard inocula (1.75 × 10^7^; *C. albicans* efg1Δ/Δ cph1Δ/Δ and *C. dubliniensis* wild-type strain Wü284) or the sub-lethal inocula (1 × 10^6^; DAY185, BWP17 + clP30, TT21, 529L, ece1Δ/Δ, and TNRG1). Animals receiving no inoculation (naïve) and lethal challenge only (1.75 × 10^7^ *Ca* + 8 × 10^7^ *Sa*) served as negative and positive controls, respectively. Mice were sacrificed at 24 h and 13 d after inoculation for collection of femur bones and processed for 4-HNE staining. (**A**) 4-HNE tissue staining by immunofluorescence (ferroptosis) at 24 h post-immunization. Tissue-associated 4-HNE is shown in green, and DAPI counterstain is shown in blue. Representative images of two repeats are shown at 40× magnification. Scale bar 100 µm. (**B**) Correlation of protection against lethal challenge and bone marrow damage (ferroptosis) 24 h post-immunization. Average percentage of protection from prior studies was plotted against mean fluorescence intensity for each isolate to determine the correlation coefficient (***R* ^2^**) and *P* value. Images and data are representative of five femur bones/inoculation groups from two repeats. (**C**) 4-HNE tissue staining by immunofluorescence (ferroptosis) of BM tissue sections comparing 24 h post-immunization using standard (1.75 × 10^7^) or sub-lethal inocula (1 × 10^6^) with *C. albicans* wild-type (DAY185, BWP17 + Clp30, TT21, and 529L) or mutant (ece1Δ/Δ and TNRG1) strains and 13 d post-immunization for the sub-lethal inocula. Femur bones were collected and processed for 4-HNE staining. BM tissue-associated 4-HNE is shown in green, and DAPI counterstain is shown in blue. Representative images of two repeats are shown at 40× magnification. Scale bar 100 µm. Images shown for 24 h sub-lethal immunization are the same as shown in frame A. (**D**) Quantification of bone marrow tissue damage (ferroptosis) 24 h and 13 d post-immunization: Tissue/cellular damage levels were assessed from tissues taken at 24 h and 13 d post-immunization by calculating 4-HNE staining (green fluorescence intensity) in each image (*n* = 10 images/inoculation group). Results are expressed as mean fluorescence intensity. Actual *P* values are listed in tables. Percentages shown in parentheses are average percentage of protection/survival following immunization and lethal challenge. (**E**) Quantification of bone marrow tissue damage (ferroptosis) 24 h post-immunization comparing standard vs sub-lethal inocula. Results are expressed as 4-HNE mean fluorescence intensity in each image (*n* = 10 images/inoculation group). Actual *P* values are listed in tables. Percentages shown in parentheses are average percentage of protection/survival following immunization and lethal challenge. WT, wild-type.

As ferroptosis can serve as a quantitative measure of tissue damage (41, 54, 55), following both standard and sub-lethal inocula immunization of the various wild-type or mutant isolates, mice were sacrificed at various times post-immunization and femurs excised and processed for BM analysis. Naïve mice, mice immunized with the standard inocula of *Cd* or *Ca* efg1Δ/Δ cph1Δ/Δ mutant, or mice given lethal challenge were included as controls. BM tissue sections were stained with anti-4-HNE antibody (green) followed by DAPI cellular staining (blue). Representative images of BM collected 24 h post-immunization with sub-lethal inocula of the various *Ca* isolates and standard inocula of the control isolates (*C. albicans* efg1Δ/Δ cph1Δ/Δ and *C. dubliniensis* wild-type strain Wü284) are shown in Fig. 4A. Gross visual assessment reveals that BM sections from naïve mice and mice immunized with the standard inocula of *Cd* or *Ca* efg1Δ/Δ cph1Δ/Δ mutant have weak 4-HNE staining, while BM sections from mice given the lethal challenge have obvious pervasive green 4-HNE staining. Levels of 4-HNE staining in BM sections of mice immunized with the sub-lethal inocula of the cadre of other *Ca* wild-type or mutant strains were variable but seemingly inversely correlated with the average level of protection following a lethal challenge (shown in parentheses); BM sections from mice immunized with *Ca* yeast-lock strain TNRG1 and wild-type *Ca* DAY185 had weak positive 4-HNE staining (60–70% protection) followed by stronger levels for all immunizing strains that did not result in appreciable protection (TT21, 529L, ece1Δ/Δ, BWP17 + Clp30; 30–40% protection). The levels of 4-HNE staining were then quantified by calculating green fluorescence intensity in a set of 10 images/inoculation group) and plotted against the average percentage of protection from lethal challenge for each immunization strain. Results in Fig. 4B reveal a significant negative correlation between fluorescence intensity (ferroptosis) and average protection against lethal challenge (*R* ^2^ = −0.773; *P* = 0.0008).

Representative images of 4-HNE staining for BM sections of mice comparing standard vs sub-lethal inocula for several of the *Ca* isolates (including isogenic parental and mutant pairs) at 24 h post-immunization and also at 13 d following sub-lethal inocula immunization (just prior to lethal challenge) are shown in Fig. 4C. Results show relatively similar 4-HNE staining between standard and sub-lethal inocula for the various isolates. By day 13 post-immunization, 4-HNE staining was relatively weak for all isolates. The quantitative analysis of the entire dataset is shown in Fig. 4D and E. BM sections from naïve mice and mice immunized with the standard inocula of *Cd* and *Ca* efg1Δ/Δ cph1Δ/Δ had the lowest levels of fluorescence intensity compared to those from the lethal challenge only control (*P* < 0.0001). Of the remaining *Ca* strains used for immunization, with the exception of TNRG1, all sections had significantly higher levels of fluorescence intensity compared to those from mice immunized with *Cd* (*P* < 0.0001). Most of the 4-HNE fluorescence intensity in bone sections from mice immunized with the various *Ca* strains were not significantly different from mice given the lethal challenge alone. The exception was *Ca* TNRG1 (yeast-lock mutant; *P* = 0.0037). Comparisons between 24 h and 13 d post-immunization show that fluorescent intensities (ferroptosis) were significantly reduced at day 13 among the various *Ca* strains, with the exception of *Ca* TNRG1, which was already low at 24 h (Fig. 4D). Comparisons between the standard and sub-lethal inocula 24 h post-immunization showed no significant differences for any isolate (Fig. 4E), in support of the similar levels of modest protection against lethal challenge between surviving mice given the standard inocula immunization and those given the sub-lethal inocula immunization (Fig. 3).

### Bone marrow architecture post-immunization with *Candida* strains as additional support of tissue damage

**Fig 5:**
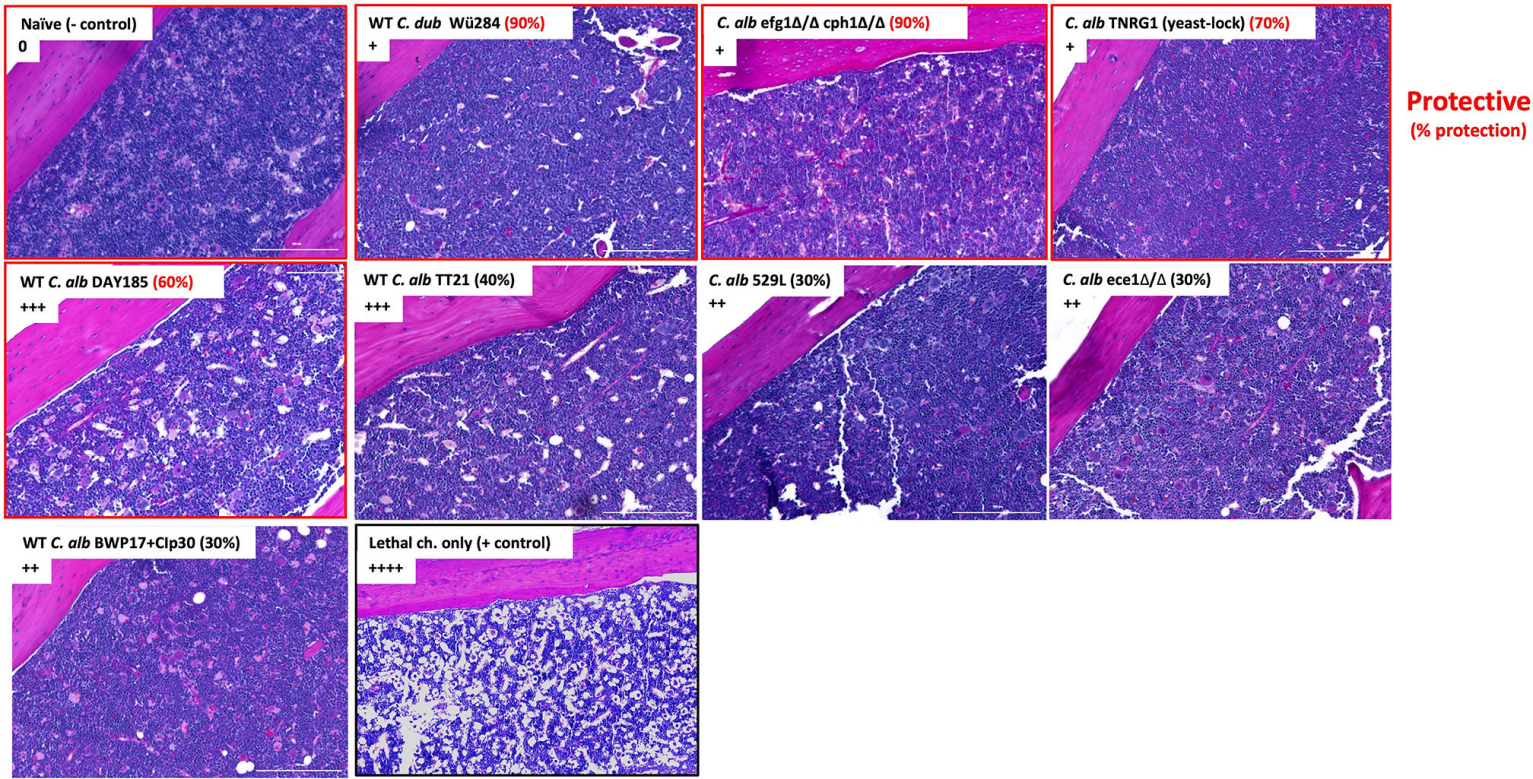
Bone marrow architecture 24 h post-immunization with *Candida* strains as additional support of tissue damage. Mice (*n* = 5) were injected i.p. with the standard inocula (1.75 × 10^7^; *C. albicans* efg1Δ/Δ cph1Δ/Δ, *C. dubliniensis* wild-type strain Wü284) or the sub-lethal inocula (1 × 10^6^; DAY185, SC5314, BWP17 + clP30, TT21, 529L, ece1Δ/Δ, and TNRG1). Animals receiving no inoculation (naïve) and lethal challenge only (1.75 × 10^7^ *Ca* + 8 × 10^7^ *Sa*) served as controls. Mice were sacrificed 24 h after inoculation for the collection of femur bones. Bones were fixed and processed for Periodic Acid-Schiff staining. Images shown were captured at 10× magnification and are representative of five femur bones/inoculation groups from two repeats. Qualitative scoring of negative space (+ to ++++ ) was conducted for all fields. Scoring is provided for each image as an overall mean score. Percentages shown in parentheses are corresponding percentage of protection/survival following immunization and lethal challenge in prior experiments. WT, wild-type. Scale bar 200 µm.

As another assessment of tissue damage in the BM following immunization, femurs were excised and from mice 24 h after sub-lethal inocula immunization with the various isolates and processed for Periodic Acid-Schiff staining of BM. Naïve mice or mice given lethal challenge were included as controls. Qualitative scoring of negative space (due to reduced cellularity or cell loss) in the BM histological sections was recorded (0 to ++++). Representative images of the histological sections and associated negative space scoring are shown in Fig. 5. Compared to dense bone histology in naïve mice (0) and mice immunized with *Cd* (+), lethal challenge results in significant negative space (++++). Mice immunized with the cadre of *Ca* wild-type or mutant strains show varying levels of bone marrow density (++ and +++), with a notable negative correlation to the average level of protection achieved following lethal challenge (noted in parentheses); femur bones that had the larger areas of negative space were from mice immunized with strains inducing the least protection. Efforts made to visualize *Candida* yeast/hyphae in the histological sections were not informative due to low levels of infiltrating organisms. Interestingly though, in other experiments designed to track infiltrating organisms in the bone marrow, we showed that GFP-labeled efg1Δ/Δ cph1Δ/Δ, albeit minimal numbers/field, were consistently shown as taken up within leukocytes (presumably macrophages) (data not shown).

## DISCUSSION

*Candida* species have an array of virulence traits that manifest during infections at a variety of anatomical sites (14, 56 – 58). This holds true as well for IAI leading to fungal/bacterial lethal sepsis. In the animal model of IAI, co-challenge of *S. aureus* with *C. albicans*, *C. kruseii*, and *C. tropicalis* all result in a lethal sepsis outcome, whereas co-challenge with *C. dubliniensis*, *C. glabrata*, and *C. auris* fail to promote lethal sepsis (23, 25, 26). Similar variability in virulence outcomes can occur for different isolates of the same species. For example, *C. albicans* wild-type strains 529L and SC5314/DAY185 are not optimal colonizers of mucosal tissues (36, 37, 45 – 48). Organ tropism can also vary between species or isolates of one species. Overall, there is a growing awareness that isolates of the same species can act quite differently in model systems, whether they be laboratory or clinical isolates. Hence, it cannot be assumed that “one strain fits all.” Rather, various strains and isolates of a particular species need to be defined by how they function/act at different anatomical sites or in different model systems.

This concept was clearly observed in the IAI model early on when we found that the virulence trait of yeast to hyphal transition for *C. albicans* was not critical to the lethal sepsis outcome when co-challenged with *S. aureus* (24). Conversely, protection against lethal fungal/bacterial sepsis by prior immunization with live *Candida* species is heavily dependent on virulence; low virulent (“live attenuated”) *Candida* species (*C. dubliniensis*, *C. glabrata*, and *C. auris*) are better inducers of the protective response than more virulent *Candida species* that are usually non-lethal as a monomicrobial insult during the normal IAI observation period (5–10 d) (25, 26). This is true despite hyphal formation not serving as a key factor in the protective response; *C. dubliniensis* that forms true hyphae is as good at inducing the protection response as low virulent species that fail to form hyphae (*C. glabrata* and *C. auris*) (25, 26). This peculiar observation, together with the recognized varied virulence between various *C. albicans* isolates in other model systems (24, 34 – 39), prompted the use of the various *Ca* strains/isolates, some as wild-type/mutant pairs, to characterize their level of virulence in the IAI model as well as their ability to induce the protective response against lethal sepsis. We hypothesized that the level of host damage caused by the isolates would be a key factor in how well a particular isolate induced the protective response. Since the bone marrow is the site for the induction of the protective response (26, 59) and the fact that the intraperitoneal inoculation of fungal species infiltrates the bone marrow within 24 h (26), we focused on tissue damage in the bone marrow for correlates of protection. While immunization with any of the low virulence isolates is normally performed with the standard inocula used for lethal challenge, recognizing the potential for some late-stage mortality with several *C. albicans* isolates at the standard inocula, we also incorporated a sub-lethal inocula (17.5 × reduced − 1 × 10^6^ CFUs), that is known to result in similar levels of protection by *C. dubliniensis* (25).

The results overall were interesting and enlightening. As expected, sub-lethal inocula had little to no mortality for all the isolates/strains. Conversely, for the standard inocula some mortality was observed for several *C. albicans* isolates over the 14-d period. In most cases, the mortality began after 6 d with the majority observed after 8 d. Interestingly, while no statistical significance was noted between isolates, the highest mortality was with DAY185 and both the wild-type parental and *ece1* mutant strains. The next highest mortality was observed with 529L. Clearly, lethal sepsis from IAI-associated dissemination is not dependent on candidalysin (*ece1*) and does not discriminate against isolates that are sub-optimal for mucosal colonization.

In the case of the protection studies, immunizing with the cadre of wild-type *C. albicans* isolates (surviving mice from standard inocula, and all mice given sub-lethal inocula) resulted in a range of protection that was not statistically significant between isolates, but most often lower than that induced by *C. dubliniensis*. This was true for immunization using both the standard and sub-lethal inocula. Moreover, protection induced by corresponding mutant strains was very similar to the wild-type parental strain. This was not all that surprising recognizing that the defects often included hyphal transformation which is a non-factor in the induction of the TII response (24). Hence, the “low or avirulent” definition for these mutants does not apply to the TII response and remains inferior to *C. dubliniensis*. The exception was *C. albicans* efg1Δ/Δ cph1Δ/Δ that lacks two transcription factors crucial for morphogenesis and other pathways required for virulence and is avirulent in all model infections tested to date, including the IAI sepsis model (38, 49 – 52). Accordingly, it induced protection similar to *C. dubliniensis*. In terms of inocula and level of protection, there appeared to be an effect of inocula when visually comparing protection induced by isolates at standard (surviving mice) vs sub-lethal inocula (Fig. 2). When isolates (wild-type or mutant) were compared directly (Fig. 3), differences were identified for two mutant isolates, efg1Δ/Δ cph1Δ/Δ and ece1Δ/Δ. In these cases, the standard immunizing inocula induced better protection than the corresponding sub-lethal inocula despite similar levels of infiltration into the BM (Fig. S1). The differences for efg1Δ/Δ cph1Δ/Δ can be explained by very weak virulence or strong host antifungal activity resulting in clearance prior to inducing the TII response, despite being present at similar levels to the parental strain in the BM at 24 h. However, this explanation cannot be used for ece1Δ/Δ, which is relatively more virulent in the IAI model. Further studies will be required to assess this interesting dichotomy particularly for downstream effects on epigenetic remodeling.

The inverse correlation of protection to BM tissue damage was borne from the observation that the flushed bone marrow in non-protected mice appeared very red compared to that from protected mice, suggestive of blood vessel damage and/or RBC lysis. Initial evaluations of optical density, lactate dehydrogenase (LDH), and hemoglobin concentrations in the BM samples collected from femurs post-immunization, however, were inconclusive. Conversely, BM histology evaluating ferroptosis by 4-HNE staining (41, 54, 55, 60) and negative space indicative of problems with hematopoietic stem cell function (61 – 64) were stronger indicators of tissue damage. Indeed, BM from naïve mice and *C. dubliniensis*-immunized mice with the highest protection (controls) had low 4-HNE staining and dense histology. In contrast, BM from lethal challenge mice and mice given *C. albicans*-immunizing isolates resulting in low levels of protection had higher 4-HNE staining and reduced cellularity (negative space) at 24 h post-inoculation. Intermediate levels of 4-HNE staining and cellularity were observed for BM from mice given *C. albicans* isolates that resulted in higher levels of protection. Quantitative analysis of ferroptosis confirmed the qualitative assessment, with significant differences observed between isolates associated with high (80–90%) vs low (40–60%) levels of protection and a highly significant negative correlation (*R* ^2^ = −0.773) between ferroptosis and average levels of protection for the various isolates. Some additional interesting data included similar levels of 4-HNE staining at 24 h between standard and sub-lethal inocula of each isolate that was supported by the similar levels of infiltration into the BM at 24 h (Fig. S1), and relative resolution of tissue damage (low 4-HNE staining) by day 13 post-immunization for all the isolates. These results are in support of the early survival data for immunization with each isolate/inocula and suggest that the tissue damage in surviving mice is not permanent and can ultimately resolve for effective induction of the protective TII response in the BM and/or release/activation of the MDSCs for ultimate function.

An important aspect of these results is speculating on what mechanistically is causing the tissue damage in the BM. One might assume the damage is due to the organism based on the overall virulence attributes of *C. albicans* vs *C. dubliniensis* and highly avirulent *C. albicans* mutants. However, we hypothesize that the damage is primarily due to the host response in the BM based on several pieces of information from this study. Firstly, since relative damage is not dependent on the strong virulence attributes of hyphal formation or the presence of candidalysin, organism-mediated damage is unlikely. Secondly, the lack of any correlation of tissue damage to fungal burden in the BM would not favor organism-mediated damage. Conversely, studies that were designed to track *C. albicans* yeast form in the BM showed the majority as intracellular in leukocytes (possibly macrophages). Hence, it is more likely that the host response to the more virulent *C. albicans* is mediating the majority of damage. Future studies will test this hypothesis by evaluating cytokines and immune mediators in the BM following inoculation with the various isolates similar to what we recently reported for immunization with *C. dubliniensis* (28). In addition, a comprehensive analysis of epigenetic changes associated with the TII response in MDSCs along with correlates to *Candida* isolate-associated tissue damage are also planned.

In conclusion, our results provide strong support for the concept that levels of protection against lethal sepsis by the MDSC TII response are inversely proportional to the local tissue damage in the BM induced by the immunizing live species/isolate. The action of the various *Ca* isolates in this model revealed definitive caveats that were not always consistent with reported virulence but nonetheless were consistent with the properties of the model, both for lethal sepsis and for protection against. Moreover, while this immunization may be a potentially exciting vaccine strategy, even the use of low virulence (live-attenuated) fungal strains to immunize against polymicrobial sepsis poses significant safety concerns. However, this may be circumvented based on our recent data revealing that protection against lethal sepsis can be equally achieved by intraperitoneal immunization with abiotic cell wall components of *C. albicans* (i.e., modified β-glucan or depleted zymosan) (65). Current studies are evaluating this abiotic vaccine strategy by monitoring infiltration of such components into the BM and any correlates of tissue damage with the induction and function of the protective MDSC-mediated TII response.

## SUPPLEMENTAL MATERIAL

Figure S1 iai.00252-23-s0001.tif Bone marrow CFUs.

Fig. S1 legend iai.00252-23-s0002.pdf Full legend of Fig. S1.

Table S1 iai.00252-23-s0003.tif Candida strains used in current study.

ASM does not own the copyrights to Supplemental Material that may be linked to, or accessed through, an article. The authors have granted ASM a non-exclusive, world-wide license to publish the Supplemental Material files. Please contact the corresponding author directly for reuse.
