# Supplementary figures and images for: Protection against lethal sepsis following immunization with Candida species varies by isolate and inversely correlates with bone marrow tissue damage
Source: Infect Immun. 2023 Sep 13;91(10):e00252-23. doi: 10.1128/iai.00252-23 (PMC10580931; doi:10.1128/iai.00252-23)

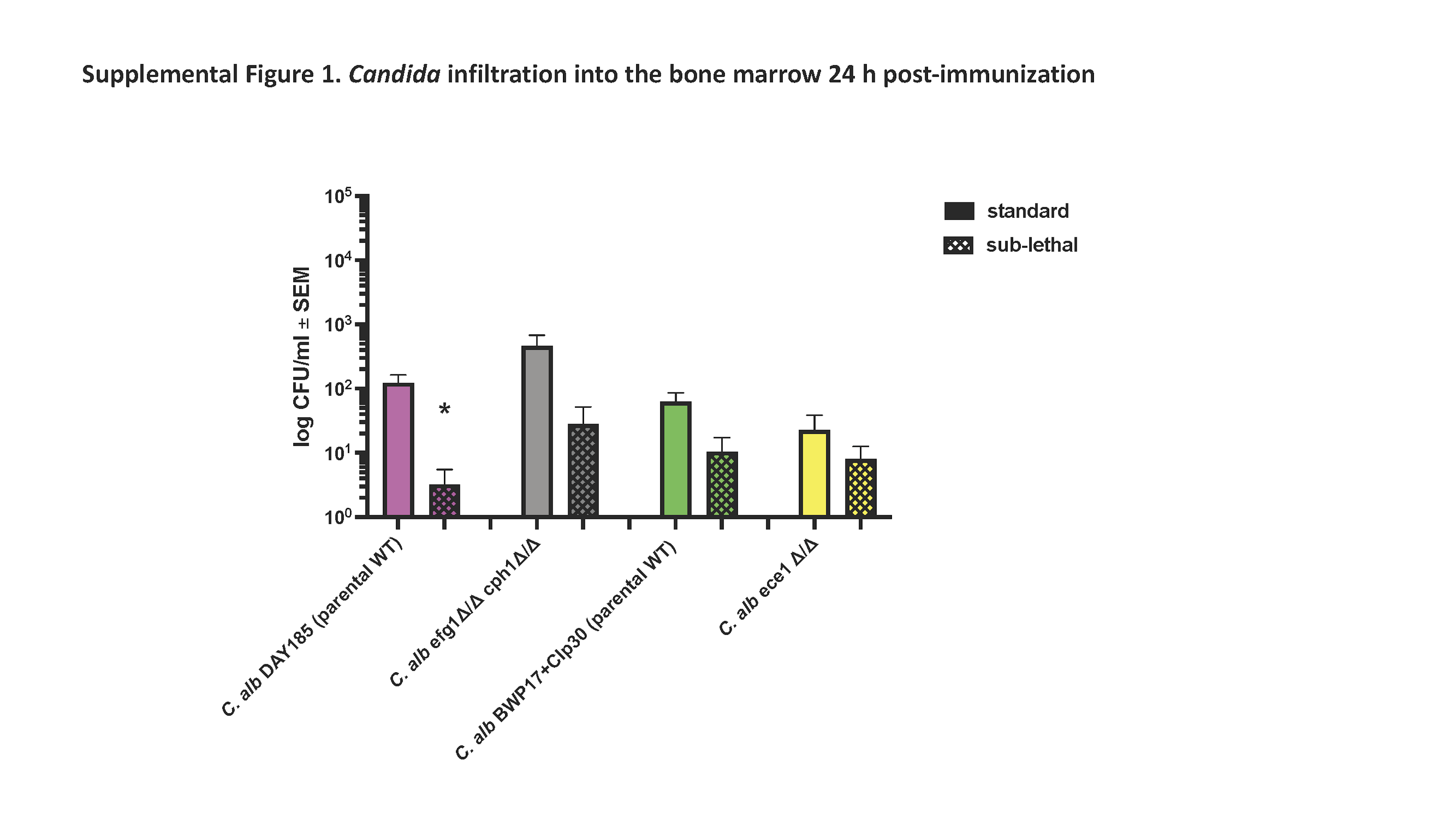

Supplement: Figure S1 — Bone marrow CFUs. [file iai.00252-23-s0001.tif]

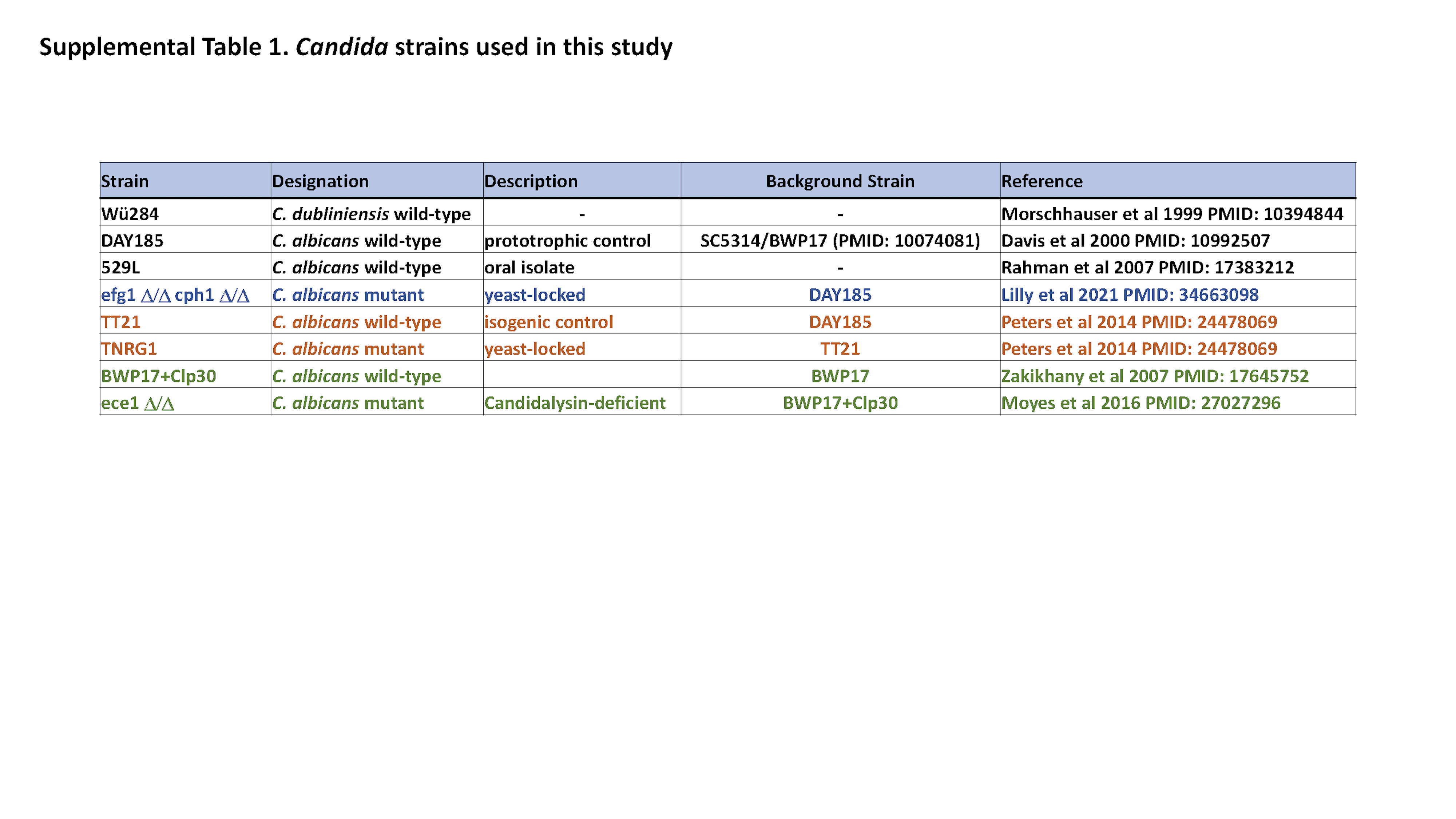

Supplement: Table S1 — Candida strains used in current study. [file iai.00252-23-s0003.tif]
