# Supplementary material for: Protection against lethal sepsis following immunization with Candida species varies by isolate and inversely correlates with bone marrow tissue damage
Source: Infect Immun. 2023 Sep 13;91(10):e00252-23. doi: 10.1128/iai.00252-23 (PMC10580931; doi:10.1128/iai.00252-23)
Supplement: Fig. S1 legend — Full legend of Fig. S1. [file iai.00252-23-s0002.pdf]

Supplemental Figure 1. Bone marrow infiltration 24 h post-immunization. Mice (n=4) were injected i.p. with the standard inocula ($1.75x10^7$) or the sub-lethal inocula ($1x10^6$) with parental wild-type strains (C. albicans DAY185; BWP17+Clp30) or corresponding mutant strains (*C. albicans* efg1Δ/Δ cph1Δ/Δ; ece1Δ/Δ). Mice were sacrificed 24 h after inoculation for collection of femur bones. Femoral bone marrow was isolated and assessed for fungal burden. Results from two repeats are expressed as CFU/ml of bone marrow cell suspension. Data were analyzed using the Student's *t* test. ****P<0.0001; ***P<0.001; **P<0.01; *P<0.05 (significance as compared between standard and sub-lethal immunizations). WT, wild-type
